# Refractory dry eye disease associated with Meige’s syndrome induced by long-term use of an atypical antipsychotic

**DOI:** 10.1186/s12886-020-01738-w

**Published:** 2020-12-02

**Authors:** Ji Eun Kim, Ji Won Jung

**Affiliations:** Department of Ophthalmology and Inha Vision Science Laboratory, Inha University School of Medicine, Incheon, South Korea

**Keywords:** Meige’s syndrome, Blepharospasm, Dry eye disease, Atypical antipsychotics, Blonanserin

## Abstract

**Background:**

We report a case of Meige’s syndrome induced by an atypical antipsychotic (blonanserin) that presented with refractory dry eye disease.

**Case presentation:**

A 37-year-old woman with a 6-month history of foreign body sensation in the eyes and difficulty in opening her eyes was treated at a local clinic for dry eye disease. Despite this treatment, her symptoms did not improve and she was transferred to our attention. Our assessment revealed involuntary movements of her eyelids accompanied by repetitive pursing of her lips. She had been undergoing treatment with blonanserin for 5 years for schizophrenia. She was diagnosed with drug-induced Meige’s syndrome after a psychiatric and neurological consultation. After a 2-month gradual dose reduction and discontinuing blonanserin, involuntary movements of the eyelids with oromandibular dystonia were resolved. Three months after discontinuing blonanserin, there was no recurrence of symptoms, and she had no exacerbation of psychotic symptoms.

**Conclusions:**

In patients with refractory dry eye disease, especially those with involuntary movements of the eyelids with oromandibular dystonia, it is important to ask about their psychotropic medications and to consider the possibility of drug-induced Meige’s syndrome and discontinuation of medications, if possible.

## Background

Dry eye disease presents with various symptoms such as irritation, stinging, dryness, blurry vision, and photophobia. Some patients with dry eye disease complain of involuntary movements of the eyelids. Foreign body sensation, photophobia, and other dry eye disease symptoms make patients frequently close their eyes. Some patients find it difficult to keep their eyes open even after treatment for dry eye disease [1].

Blepharospasm associated with spasm of other facial or oromandibular muscles is considered Meige’s syndrome [2]. The exact etiologies are unknown, but the hypothesis related to dopaminergic and cholinergic hyperactivity is the most widely accepted [3]. The causes of Meige’s syndrome are unclear, but it is commonly induced by medications: psychotropics, antiemetics (such as metoclopramide), dopamine agonists (such as levodopa and bromocriptine), antidepressants (such as selective serotonin reuptake inhibitors) and antihistamines [4].

Tsubota et al. [1] reported that among 325 patients with dry eye disease, 57% (28/49) of those who unresponsive to conventional treatment were diagnosed with Meige’s syndrome. We report a case of a patient with refractory dry eye disease who was diagnosed with Meige’s syndrome induced by long-term use of an atypical antipsychotic (blonanserin).

## Case presentation

A 37-year-old woman complained of foreign body sensation in the eyes and difficulty in opening her eyes for 6 months. The involuntary movements of her eyelids worsened, and she could not keep her eyes open. At the local clinic, she was diagnosed with refractory dry eye disease because artificial tears and other conventional dry eye treatments more than 6 months were ineffective. Intense pulsed light (IPL) was attempted once at the local clinic, but there was no improvement in her symptoms. She had been treated for schizophrenia for 5 years with blonanserin and had no other medical histories.

On ocular examination, her corrected visual acuity was 20/20 in both eyes. There were no specific findings, except scattered micropunctate corneal staining in the inferior area on slit-lamp microscopy. Schirmer’s test was performed at 7 mm/5 min in the right eye and 10 mm/5 min in the left eye. The ocular surface staining score was 1/33 in both eyes (National Eye Institute grading scale, 0–33), and the tear breakup time (TBUT) was 4 s for both eyes. Her ocular surface disease index (OSDI) questionnaire score was 80.00, and stage 1 meibomian gland dysfunction (MGD) was noted. According to the dry eye severity grading system (Dry eye workshop (DEWS))[5], the patient’s sign was of level 2–3 severity and the symptoms of dry eye disease were of level 3–4 severity. Hence, the patient was diagnosed with moderate-to-severe dry eye disease.

**Fig. 1:**
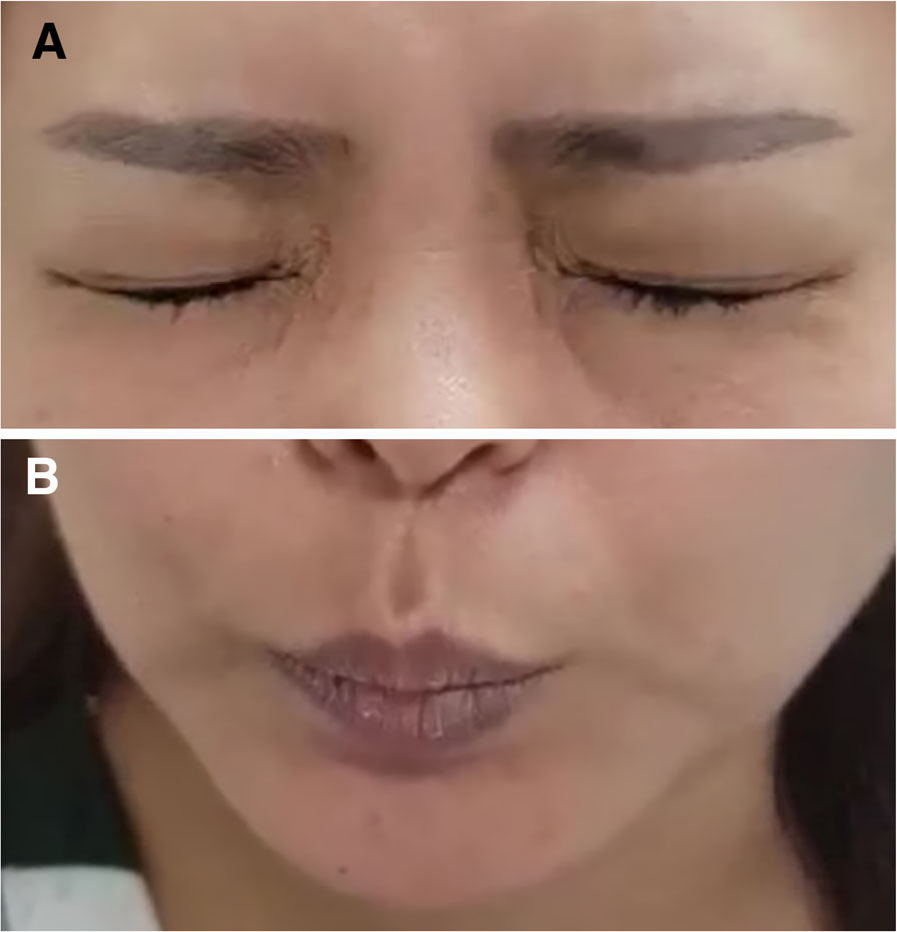
The patient showed the symmetric and involuntary movements of the eyelids accompanied by pursing of her lips, which was presumed to be oromandibular dystonia

Extraocular eye movements were normal, and there was no evidence of nystagmus or relative afferent pupillary defect. However, she showed symmetric and involuntary movements of the eyelids accompanied by pursing of the lips, which was presumed to be oromandibular dystonia (Fig. 1).

We consulted the neurology department regarding the involuntary movements of the eyelids with oromandibular dystonia, and no abnormal findings were revealed on brain magnetic resonance imaging/angiography. Electromyography test revealed that bilateral facial nerve conduction and blink reflex were normal. The lateral spread response test was positive at the orbicularis oculi muscle when the mandibular branch of the facial nerve was stimulated.

We consulted the psychiatry department for the suspicion of drug-induced Meige’s syndrome. The dose of blonanserin was reduced and the anticholinergic (procyclidine) and the anticonvulsant (clonazepam) were added to relieve the blepharospasm. Two months after dose reduction and discontinuing blonanserin treatment, involuntary movements of the eyelids with oromandibular dystonia were completely resolved. Procyclidine and clonazepam were discontinued after short-term administration, because her symptoms resolved after discontinuing blonanserin.

Three months after discontinuing blonanserin treatment, there was no recurrence of involuntary movements of the eyelids with oromandibular dystonia, and she had no exacerbation of psychotic symptoms. Her dry eye symptoms were alleviated (OSDI: 24.00) and the TBUT was 6 s for both eyes, and the other ocular surface status parameters did not change.

## Discussion and conclusions

We have reported a case of a relatively young woman, who was referred to our hospital because of refractory dry eye disease despite conventional dry eye treatments for more than 6 months. Dry eye treatment is aimed at minimizing inflammation and stabilizing the tear film. Artificial tears are essential for the treatment of dry eyes, and administration of topical anti-inflammatory drugs are helpful for more severe cases. Autologous serum tears, contact lenses, and punctal occlusion are used for refractory dry eye disease. In-office treatment, such as IPL, have been recently used for dry eyes with MGD [6]. The patient had a moderate-to-severe refractory dry eye disease, and IPL therapy was attempted because conventional dry eye treatments for more than 6 months were ineffective. However, after the first IPL therapy, there was no improvement in dry eye symptoms; therefore no additional IPL was performed.

We focused on the involuntary movements of her eyelids with oromandibular dystonia, which were different from other dry eye symptoms. Therefore, we consulted the neurology department for further evaluation of her symptoms, but no abnormal neurological findings were revealed. In addition, we enquired about use of systemic medications; initially, she was hesitant to talk about her psychiatric disease and medication; however, she finally disclosed using antipsychotics.

Meige’s syndrome is a type of cranial dystonia characterized by a combination of blepharospasm and oromandibular dystonia [4]. The diagnosis of Meige’s syndrome is mainly clinically based and easy to miss. The etiology of Meige’s syndrome is related to the upper brain stem and abnormal basal ganglia dopamine receptor hypersensitivity, hyperactive cholinergic nervous system, and low gamma-amino butyric acid function [7]. The causes of Meige’s syndrome are not well known; however, it is commonly induced by drugs such as antipsychotics and levodopa; and it is rarely induced by cerebellar degeneration, basal ganglia dysfunction, and brain tumors [8].

Several cases of Meige’s syndrome induced by typical antipsychotics have been reported. Atypical antipsychotics cause few extrapyramidal symptoms; therefore few cases of Meige’s syndrome have been reported [9–13]. In most cases, symptoms improve after discontinuing or switching the treatment with the causative drug. We reviewed the cases of Meige’s syndrome caused by atypical antipsychotics (Table 1). Also in our case, the patient’s symptoms improved approximately 2 months after gradual dose reduction and discontinuing blonanserin treatment in cooperation with a psychiatrist. Blonanserin is a novel atypical antipsychotic with specific receptor binding characteristics on dopamine D2 and D3, and serotonin 5-HT2A receptors as antagonists. This drug has a greater affinity for D2 receptors, which are responsible for the development of dyskinesia [14]. A few cases of blonanserin-induced oromandibular dystonia have been reported; [15] however, our case is the first case of blonanserin-induced Meige’s syndrome.

**Table 1:** Review of studies of Meige’s syndrome induced by atypical antipsychotics

Study	Cause	Treatment	Outcome
Umene-Nakano et al. [9]	Aripiprazole	Exchange to Perospirone	Symptoms improved facial movement not completely disappeared
Takahashi et al. [10]	Perospirone	Perospirone reduction & add Biperidine	Symptoms improved facial movement not completely disappeared
Kiliç et al. [11]	Olanzapine Quetiapine	Exchange to Diazepam	Blepharospasm markedly improved
Yoshimura et al. [12]	Risperidone	Exchange to Paliperidone	Symptoms completely remitted
Marques et al. [13]	Paliperidone	Exchange to Clozapine	Meige’s syndrome had disappeared

In conclusion, in refractory dry eye disease patients, especially in those who show involuntary movements of the eyelids with oromandibular dystonia, it is important to ask about the use of psychotropic medications and to consider the possibility of drug-induced Meige’s syndrome and discontinuation of medications, if possible.

## Data Availability

All data generated or analysed during this study are included in this published article.

## Abbreviations

IPL: Intense pulsed light
TBUT: Tear breakup time
OSDI: Ocular surface disease index
MGD: Meibomian gland dysfunction
